# Gender Differential Transcriptome in Gastric and Thyroid Cancers

**DOI:** 10.3389/fgene.2020.00808

**Published:** 2020-07-30

**Authors:** Abel Sousa, Marta Ferreira, Carla Oliveira, Pedro G. Ferreira

**Affiliations:** ^1^Instituto de Investigação e Inovação em Saúde da Universidade do Porto, Porto, Portugal; ^2^Institute of Molecular Pathology and Immunology of the University of Porto, Porto, Portugal; ^3^Graduate Program in Areas of Basic and Applied Biology, Abel Salazar Biomedical Sciences Institute, University of Porto, Porto, Portugal; ^4^European Molecular Biology Laboratory, European Bioinformatics Institute, Wellcome Genome Campus, Cambridge, United Kingdom; ^5^Faculty of Medicine of the University of Porto, Porto, Portugal; ^6^Department of Computer Science, Faculty of Sciences of the University of Porto, Porto, Portugal; ^7^Laboratory of Artificial Intelligence and Decision Support, Institute for Systems and Computer Engineering, Technology and Science, Porto, Portugal

**Keywords:** gastric cancer, thyroid cancer, gender differences, RNA-seq, The Cancer Genome Atlas, Genotype-Tissue Expression, differential expression, gene co-expression networks

## Abstract

Cancer has an important and considerable gender differential susceptibility confirmed by several epidemiological studies. Gastric (GC) and thyroid cancer (TC) are examples of malignancies with a higher incidence in males and females, respectively. Beyond environmental predisposing factors, it is expected that gender-specific gene deregulation contributes to this differential incidence. We performed a detailed characterization of the transcriptomic differences between genders in normal and tumor tissues from stomach and thyroid using Genotype-Tissue Expression (GTEx) and The Cancer Genome Atlas (TCGA) data. We found hundreds of sex-biased genes (SBGs). Most of the SBGs shared by normal and tumor belong to sexual chromosomes, while the normal and tumor-specific tend to be found in the autosomes. Expression of several cancer-associated genes is also found to differ between sexes in both types of tissue. Thousands of differentially expressed genes (DEGs) between paired tumor–normal tissues were identified in GC and TC. For both cancers, in the most susceptible gender, the DEGs were mostly under-expressed in the tumor tissue, with an enrichment for tumor-suppressor genes (TSGs). Moreover, we found gene networks preferentially associated to males in GC and to females in TC and correlated with cancer histological subtypes. Our results shed light on the molecular differences and commonalities between genders and provide novel insights in the differential risk underlying these cancers.

## Introduction

Sexual dimorphism is a taxonomically widespread phenomenon, whereby certain traits differ consistently between males and females within a given species. In humans and other animals, these differences go beyond morphological and behavioral traits and include molecular phenotypes such as gene expression (Trabzuni et al., 2013; Melé et al., 2015; Gershoni and Pietrokovski, 2017; Naqvi et al., 2019). It has been hypothesized that sex-specific gene regulation underlies important phenotypic gender differences and may contribute to gender differential susceptibility to disease (Ober et al., 2008; Rawlik et al., 2016; Labonté et al., 2017). Cancer has a considerable differential incidence between genders (Dorak and Karpuzoglu, 2012; Ali et al., 2016; Clocchiatti et al., 2016), with men showing a higher cancer incidence than women in 32 of 35 anatomical sites (Edgren et al., 2012). In 13 of these sites, the differences could not be explained by known risk factors, including smoking, alcohol consumption, and potential occupational carcinogens such as toxic metals and ionizing radiation. Men are at higher risk and worst prognosis in several types of cancers in non-reproductive tissues, including skin, esophagus, stomach, liver, and urinary bladder cancers (Siegel et al., 2018). One remarkable exception is the thyroid tissue, where women have three times higher risk of developing cancer (Rahbari et al., 2010). For malignancies such as acute lymphoblastic leukemia or non-Hodgkin lymphoma, the gender-bias incidence occurs already in childhood, being more common in boys (Dorak and Karpuzoglu, 2012). Although environmental and lifestyle factors largely contribute to gender disparities in cancer, it seems clear that gender intrinsic molecular factors may also play an important role.

Cancer sexual disparity may be the consequence of a complex interplay between sex chromosomes and the hormonal system (Clocchiatti et al., 2016). In females, several X chromosome genes may escape the XIST-dependent inactivation, triggering an imbalanced expression between genders (Carrel and Willard, 2005). This asymmetry can make females more resistant to inactivating mutations in tumor-suppressor genes (TSGs) (Dunford et al., 2017). For instance, *UTX* is known to escape silencing in females (Bellott et al., 2014) and to have inactivating mutations in renal and esophageal cancers, more prevalent in males (van Haaften et al., 2009). Sex steroid hormones can interact with the cellular receptors estrogen receptor-α (ERα), ERβ, and androgen receptor (AR) and induce gene expression changes, affecting cellular metabolic states, tumor microenvironments, and the immune system (Clocchiatti et al., 2016). For example, in liver cancer, more frequent in males, AR stimulates and ERα restrains cellular proliferation (Li et al., 2012). Moreover, an estrogen-mediated inhibition of inflammatory IL-6 production may reduce liver cancer risk in females (Naugler et al., 2007). In thyroid cancer (TC), the association between sex hormones and cancer risk is uncertain (Rahbari et al., 2010). While animal models and *in vitro* studies suggest that sex hormone levels can affect TC tumorigenesis and progression, the same has not been observed at the clinical level (Yao et al., 2011). Sex hormones are also known to regulate the thyroid gland in a gender-specific manner (Banu et al., 2002). It is therefore possible that the thyroid glands in females are biologically more prone to cancer development than in males (Yao et al., 2011).

Pan-cancer systematic studies on gender differences have identified sex-biased genes (SBGs) and pathways across several cancer types from The Cancer Genome Atlas (TCGA) project (Ma et al., 2016; Yuan et al., 2016). These studies found that sex-specific gene signatures have differential responses to chemical and genetic agents and that, in certain cancer types, more than 50% of clinically relevant genes are differentially expressed between sexes. Importantly, while TC showed extensive sex-biased gene expression, the gender differences of gastric cancer (GC) remained uncharacterized.

In this work, we set out to provide a fine-detailed characterization of the gender differential transcriptome in GC and TC (Cancer Genome Atlas Research Network, 2014a, b), chosen due to their clear unbalanced gender incidence. While GC is two times more common in males, TC is three times more common in females (Siegel et al., 2018). Our results demonstrate that sex-biased gene expression is more pronounced in normal tissues than tumor tissues and that most of the shared variation arises from the sexual chromosomes. Expression of several cancer-associated genes differs between genders, with TSGs preferentially downregulated in the tumor tissue of the most susceptible gender. Gene co-expression network analysis revealed an extensive topological preservation between genders, with gender-specific networks appearing correlated with cancer histological subtypes.

## Materials and Methods

### Data Collection

Gene-level TCGA mRNA-seq data for GC and TC tumor matched-normal samples were downloaded from the Genomic Data Commons (GDC) data portal, and from the Genotype-Tissue Expression (GTEx) portal for stomach and thyroid normal samples, in reads per kilobase of exon model per million mapped reads (RPKM) and read counts formats. TCGA methylation data as beta values per probe were obtained from the FireBrowse portal.

### Data Preprocessing

Protein-coding and long intervening/intergenic non-coding RNA (lincRNA) genes were selected for downstream analysis. After removing lowly expressed genes (less than 5 counts per million in at least 20% of samples), the TCGA datasets comprised 12,690 genes for TC and 13,674 genes for GC. The GTEx datasets comprised 12,501 genes for thyroid and 12,371 genes for stomach. The TCGA and GTEx samples yielded a total of 11,734 genes for thyroid and 11,842 genes for stomach. Principal component analysis (PCA) analysis was then performed using *prcomp* function in R.

### Differential Gene Expression

**FIGURE 1:**
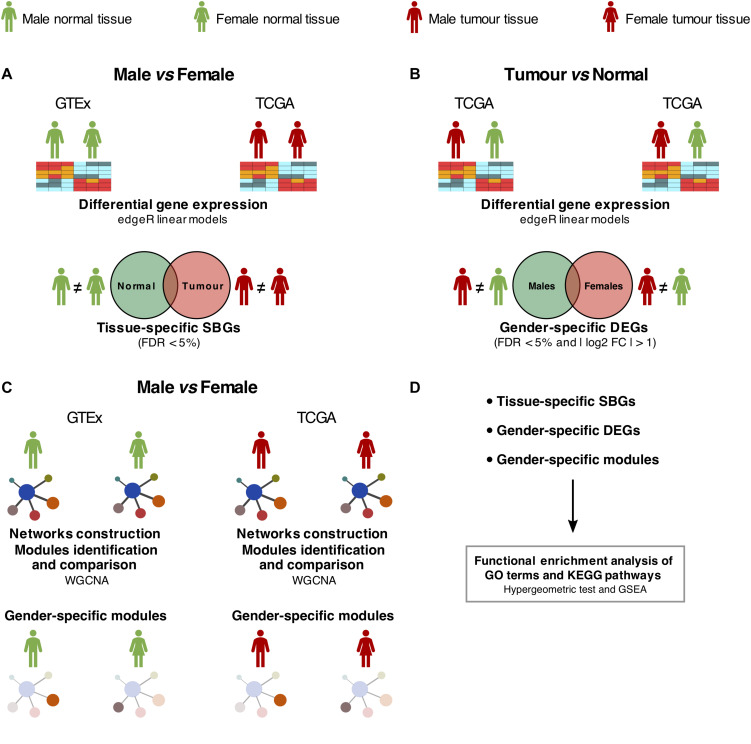
Study design. **(A)** Differential expression analysis between males and females in normal [Genotype-Tissue Expression (GTEx)] and tumor [The Cancer Genome Atlas (TCGA)] samples, adjusted for confounding effects. SBGs, sex-biased genes. **(B)** Differential expression analysis between tumor and matched-normal (TCGA) samples in males and females, adjusted for confounding effects. DEGs, differentially expressed genes. **(C)** Differential co-expression network analysis between males and females in normal (GTEx) and tumor (TCGA) samples. **(D)** Functional enrichment analysis of SBGs, DEGs, and gene co-expression modules was performed using hypergeometric-based tests and Gene Set Enrichment Analysis (GSEA).

Differential expression analysis was performed using the edgeR package (Robinson et al., 2010) for the comparisons represented in Figures 1A,B and taking into account several covariates (Supplementary Methods).

We selected genes differentially expressed between genders using a false discovery rate (FDR) threshold of 5%, additionally requiring for tumor and matched-normal comparison an absolute log2 fold change higher than 1. Tissue-specific SBGs and gender-specific tumor-normal biased genes were calculated by intersecting both gene sets.

### Differential Gene Promoter Methylation

The differential methylation analyses were performed using a Wilcoxon rank sum test (*wilcox.test* R function). Differentially methylated genes were defined with an FDR < 5%. See Supplementary Methods for more details.

### Gender Differential Co-expression Network Analysis

We built gene co-expression networks for each gender, in TCGA tumor and GTEx normal samples, for stomach and thyroid tissues, using the Weighted Correlation Network Analysis (WGCNA) package (Langfelder and Horvath, 2008). We compared male to female networks by calculating the percentage of overlapping genes between each pair of modules, where each module belongs to a different network. Based on the overlap percentage and on the Fisher’s exact test *P*-value, modules were classified as gender-specific, lowly preserved, moderately preserved, and highly preserved between genders.

### Association of Gender-Specific Modules With Cancer Clinical Traits

We evaluated the biological significance of gender-specific modules in tumors by fitting a linear regression model between the module eigengene as dependent variable and the cancer clinical traits overall survival (in days), tumor stage [American Joint Committee on Cancer (AJCC) staging system], and cancer histological subtype as independent variables (Supplementary Methods). For modules associated with cancer histological subtypes, we selected the hub genes and tested them for differential expression between histological subtypes using a Kruskal–Wallis rank sum test (*kruskal.test* R function).

### Functional Enrichment Analysis

We performed functional enrichment using hypergeometric tests and Gene Set Enrichment Analysis (GSEA), implemented in the functions *enrichr* and *GSEA* from the *clusterProfiler* package (Yu et al., 2012). We used gene sets downloaded from the MSigDB database (Supplementary Methods). The enrichment for TSGs, oncogenes, X-inactivated genes, and differentially methylated genes was performed using the *fisher.test* R function (*alternative* = *“greater”*).

## Results

### Gender Differences Are Not Revealed by Genome-Wide Transcriptomic Profiles

We analyzed RNA-seq data, generated by the TCGA project, of 375 GC samples (female, *n* = 134 and male, *n* = 241) and 502 TC samples (female, *n* = 367 and male, *n* = 135). As normal tissue counterparts, we used data from GTEx V6 (GTEx Consortium, 2013) that encloses 225 normal stomach samples (female, *n* = 82 and male, *n* = 111) and 381 normal thyroid samples (female, *n* = 112 and male, *n* = 211), as well as the TCGA tumor-matched normal samples (stomach, *n* = 32 and thyroid, *n* = 58). Altogether, we collected 1,483 tumor and normal samples from stomach and thyroid tissues (Supplementary Figure S1A; see section “Materials and Methods”). Minimal expression filtering yielded 11,842 genes in stomach and 11,734 in thyroid (see section “Materials and Methods”).

Principal component analysis analysis revealed that both tissues segregated by dataset of origin rather than tumor or normal status (Supplementary Figure S1B). As a consequence of this strong batch effect, all analyses have been performed separately for the TCGA and the GTEx datasets. No global distinct transcriptomic patterns were observed between genders (Supplementary Figure S1B). Confounding effects were successfully regressed out (Supplementary Figures S1C, S2).

A detailed characterization of the transcriptomic differences between genders was performed following the design in Figure 1.

### Tumor and Normal Tissues Show Specific Sex-Biased Genes

**FIGURE 2:**
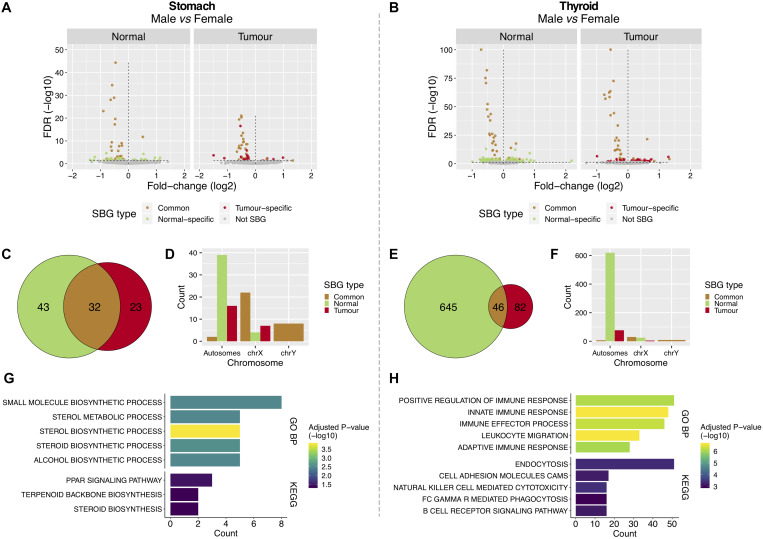
Features of sex-biased genes in tumor and normal stomach and thyroid tissues. **(A,B)** Differentially expressed genes between males and females in normal [Genotype-Tissue Expression (GTEx)] and tumor [The Cancer Genome Atlas (TCGA)] tissues from the stomach and thyroid. The Y-chromosome genes and *XIST* were removed for visualization purposes. Vertical lines: left, genes over-expressed in females (under-expressed in males); right, genes under-expressed in females (over-expressed in males). **(C,E)** Shared and tissue-specific sex-biased genes (SBGs). **(D,F)** Distribution of SBGs in autosomes and sexual chromosomes. **(G,H)** Gene Ontology (GO) biological processes (GO BPs) and Kyoto Encyclopedia of Genes and Genomes (KEGG) pathways enriched in the normal-specific SBGs [top 5; false discovery rate (FDR) < 5%].

To understand the gender- and tissue-specific (tumor and normal) expression patterns in stomach and thyroid, we performed gender differential expression analysis using the normal samples from both tissues available from GTEx, followed by the same analysis in the tumor samples from TCGA (Figures 1A,D). In the stomach, we found 75 SBGs in the normal and 55 SBGs in the tumor, of which 32 were common (Figures 2A,C and Supplementary Table S1). For the thyroid, we found 691 and 128 SBGs in the normal and tumor, respectively, with 46 genes in common (Figures 2B,E and Supplementary Table S2). Common SBGs originated mostly from the X and Y chromosomes (Figures 2D,F) and were similar in the stomach and thyroid (27 genes; 84 and 59% of the common SBGs in the stomach and thyroid). These genes were involved in translational initiation, protein dealkylation, and demethylation, with preferential location in the genomic regions chrXp22/p11/q13 and chrYq11 (Supplementary Figure S3; FDR < 5%). Contrarily, normal and tumor-specific gender differences derived mostly from autosomes (Figures 2D,F).

The 43 normal-specific SBGs in the stomach (Figure 2C) were enriched for sterols metabolic processes and in the peroxisome proliferator-activated receptor (PPAR) signaling pathway (Figure 2G; FDR < 5%). The majority of genes involved in these processes were over-expressed in the normal stomach of females, the less affected gender in GC (Supplementary Figure S4A). In the thyroid, the 645 normal-specific SBGs (Figure 2E) were enriched in innate and adaptive immune response processes and lipid metabolism (Figure 2H and Supplementary Figure S5A; FDR < 5%), including over-expression in females, the most affected gender in TC (Supplementary Figures S4B, S5B).

The 23 tumor-specific SBGs in GC and 82 in TC (Figures 2C,E) were involved in lipid metabolic processes (Supplementary Figure S6; FDR < 20%).

The X-chromosomal SBGs from GC and TC showed an enrichment for genes that escape X-inactivation (Supplementary Table S3; *P*-value < 5%). Of note, *USP9X* (log2FC/FDR = −0.31/1.7e-06), a previously reported cancer driver, *TXLNG* (−0.54/3.3e-17), *OFD1*, *MED14*, and *CDK16* are known to evade X-inactivation and were over-expressed in females’ GC. This suggests that tumorigenesis in females’ stomach may take advantage from over-expression of genes that escape X-inactivation.

Gender differential promoter methylation analysis showed that 54 (GC) and 20% (TC) of the previously found SBGs were differentially methylated (Supplementary Figures S7A,B; see section “Materials and Methods”; *P*-value = 2e-15, 2.7e-6). Among these, 96% belong to the X-chromosome and 89% are known to escape X-inactivation.

### Tumor Suppressor Genes Show Tumor-Specific Under-Expression in the Susceptible Gender

**FIGURE 3:**
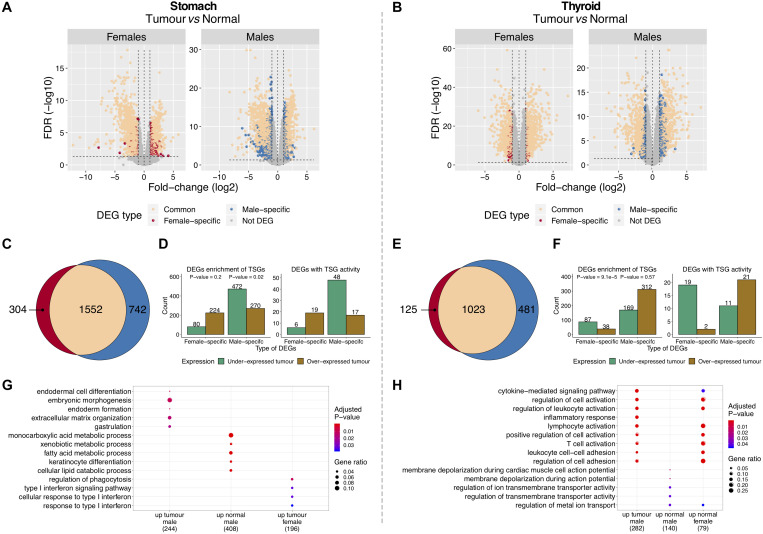
Features of differentially expressed genes (DEGs) between tumor and normal tissues in gastric cancer (GC) and thyroid cancer (TC). **(A,B)** DEGs between tumor and matched-normal samples in females and males. Vertical line (*x* = 0): left, genes under-expressed in tumors (over-expressed in normal); right, genes over-expressed in tumors (under-expressed in normal). **(C,E)** Shared and gender-specific DEGs. **(D,F)** Gender-specific DEGs over- and under-expressed in tumors. Left, all DEGs and respective enrichment for tumor-suppressor genes (TSGs) (Fisher test *P*-value). Right, DEGs with TSG activity. **(G,H)** Gene Ontology (GO) biological processes significantly enriched and shared between gender-specific DEGs (male/female) over-expressed in tumor and normal tissues (up) [false discovery rate (FDR) < 5%].

To identify tumor-normal differentially expressed genes (DEGs) in each gender, tumor and matched-normal TCGA samples were compared (Figures 1B,D and Supplementary Tables S4, S5). In GC, we found 1,552 DEGs shared between genders, corresponding to 84% of the female and 68% of the male DEGs (Figures 3A,C). Similarly, in TC, 89% of the female and 68% of the male DEGs were common to both genders (1,023 DEGs) (Figures 3B,E). The shared DEGs likely reflect genes that, independently of the gender, are pivotal for tumorigenesis. In fact, a significant proportion of these are oncogenes (5% for GC and 6% for TC; *P*-value = 0.02, 9e-3). In GC, it includes the cancer drivers *WHSC1*, *CBFB*, *RUNX1*, *EZH2* (male, female log2FC/FDR = 1.6/2.1e-10, 1.9/6e-7), *MET*, and *CARD11*. In TC, we recapitulated *MET* (2.6/2.2e-10, 2.5/3.1e-25) and *RUNX1*, plus *CCND1*, *CDKN1A*, *ERBB3*, *FOXQ1*, *FGFR3*, and the known oncogene in TC *ZCCHC12* (Wang et al., 2017).

Gender-specific DEGs were frequently under-expressed in tumors of the most susceptible sex (males in GC and females in TC), but not in the less susceptible one [Figures 3D,F (left plots)]. Of 742 male-specific DEGs in GC and 125 female-specific DEGs in TC, 64 and 70% were under-expressed in tumors, respectively. Following this trend, we found a significant enrichment for TSGs on males in GC (9%) and females in TC (17%) [Figures 3D,F (left plots)], with the majority being under-expressed in tumors [Figures 3D,F (right plots)].

Overall, our results showed that for these two cancers, the majority of tumor-normal DEGs were shared by genders and have oncogenic properties. Most of the gender-specific DEGs were under-expressed in tumors from the most susceptible gender with a significant fraction being TSGs.

Promoter methylation analysis between tumor and matched-normal tissues of TC (data not available for GC) showed that in females and males, 53 and 26% of the DEGs were differentially methylated (Supplementary Figures S7C,D; see section “Materials and Methods”; *P*-value = 1e-3, 1.7e-24).

### Tumor-Normal Differentially Expressed Genes Were Enriched for Functional Gene Categories

Functional enrichment analysis showed that in GC, gender-common DEGs were involved in muscle structure development and contraction, female-specific in cellular responses to cytokine stimulus and male-specific in epithelial cell differentiation and metabolic processes (Supplementary Figure S8A; FDR < 5%). In TC, gender-common DEGs were involved in positive regulation of cellular proliferation and pathways in cancer, female-specific in regulation of cell adhesion and T-cell receptor signaling pathways, and male-specific in response to cytokines and innate immune response processes (Supplementary Figure S8B; FDR < 5%).

In TC, female-specific DEGs over-expressed in normal tissues and male-specific DEGs over-expressed in tumor tissues were involved in similar processes and pathways (Figure 3H; FDR < 5%). Male-specific DEGs over-expressed in normal tissues were enriched for ion transmembrane transport activity (Figure 3H; FDR < 5%). In GC, there were no clear patterns, with gender-specific DEGs showing distinct and diverse functions (Figure 3G).

### Gender-Specific Gene Networks in Cancer Are Associated With Histological Subtypes

**FIGURE 4:**
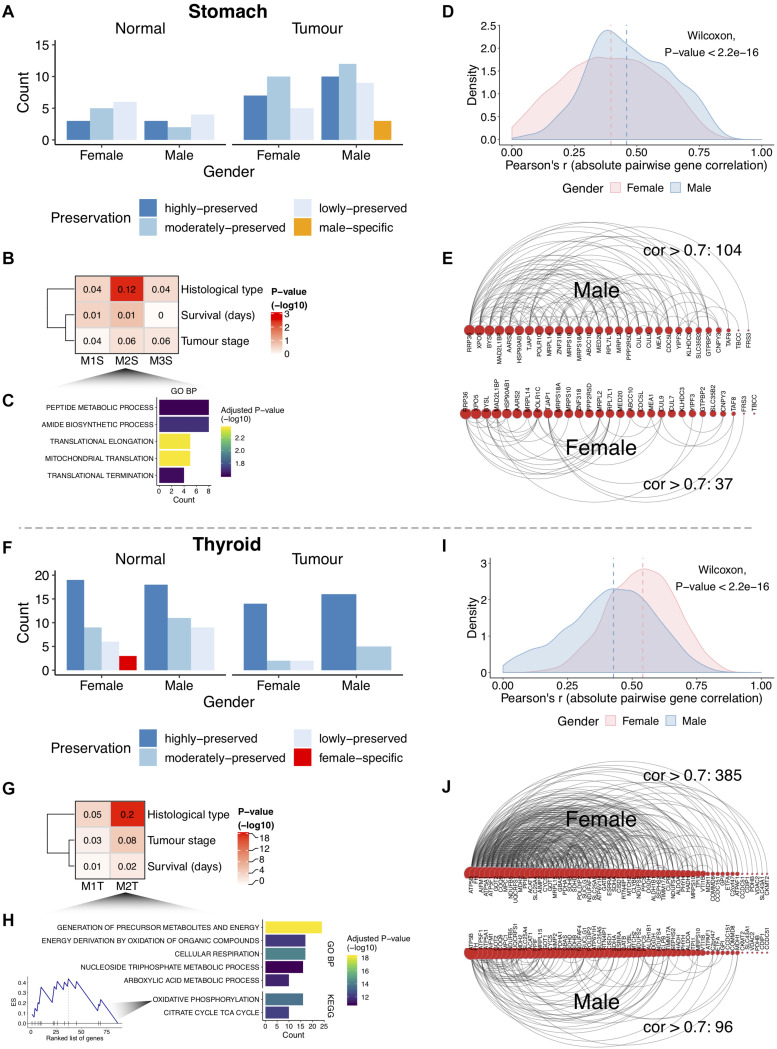
Gender differential co-expression network analysis. **(A,F)** Number of modules in stomach and thyroid tissues. **(B,G)** Association between the gastric cancer (GC) male-specific modules and the thyroid cancer (TC) female lowly preserved (in males) modules with cancer clinical traits. The numbers inside the heat maps are regression-derived *R*^2^. The one-way ANOVA-derived *P*-value is shown (–log10). Associations with survival were also tested using Cox hazard regressions (log-rank *P*-values > 5%). **(C,H)** Gene Ontology (GO) biological processes (GO BPs) and Kyoto Encyclopedia of Genes and Genomes (KEGG) pathways enriched in the GC M2S module and the TC M2T module [top 5; false discovery rate (FDR) < 5%]. Enrichment score (ES) for the oxidative phosphorylation pathway in M2T is highlighted, with genes sorted by intra-modular connectivity degree. **(D,I)** Distribution of the pairwise gene correlations (absolute Pearson’s r) for all gene pairs in M2S and M2T. Vertical lines indicate medians. **(E,J)** Arc diagrams representing gene pair correlations for M2S (29 genes from 45) and M2T (61 genes from 86). Arcs represent gene pair correlations >0.7. Genes are sorted by number of connections. The gene pairs with correlations >0.7 were selected in the gender where the module shows specificity (males in M2S and females in M2T). The number of correlations >0.7 decreases in the opposite gender.

Co-expression network analysis identifies groups of genes, called network modules, coherently expressed across samples. Such modules may highlight biologically related genes. We reasoned that beyond single gene sex-biased expression, there are differences between genders regarding the coordinated expression of groups of genes for tumor and normal tissues (Figures 1C,D). After removing possible confounding effects (Supplementary Figures S1C, S2), a full gene co-expression network was built and modules were identified for each gender (Supplementary Figures S9, S10; see section “Materials and Methods”). For the stomach, we found 23 modules in normal tissue (female: 14; male: 9) and 56 in tumors (female: 22; male: 34) (Supplementary Figure S11A). In the thyroid, we found 75 modules in normal tissue (female: 37; male: 38) and 39 in tumors (female: 18; male: 21) (Supplementary Figure S11A). The number of genes inside modules ranged from 21 to 5,976, with a median size of 119 genes per module (Supplementary Figure S11B). Next, modules were compared between genders in terms of their overlap and deemed as preserved (lowly, moderately, or highly) or gender-specific (Supplementary Figure S12; see section “Materials and Methods”). Most modules were preserved in tumor and normal tissues (Figures 4A,F). Three female-specific modules were found in normal thyroid related to vasculature development and angiogenesis, thyroid hormone, and sterol metabolism (Supplementary Figure S13).

Consistent with the higher sex-biased cancer incidence, we found three male-specific modules in GC and two female lowly preserved modules (in males) in TC (Figures 4A,F). Correlation of the modules representative expression profile with the cancer clinical traits (see section “Materials and Methods”) revealed one GC module (M2S, *P*-value = 4e-3) and one TC module (M2T, 4e-18) associated with the cancer histological subtypes (Figures 4B,G). The former (M2S) involved genes related to peptide metabolism and translation elongation (Figure 4C; FDR < 5%). The latter (M2T) was related to cellular respiration processes, with the most highly connected (hub) genes forming part of the oxidative phosphorylation pathway (Figure 4H; FDR < 5%). A higher intra-module correlation was found for the gender where the module is specific (Figures 4D,I; *P*-value < 2.2e-16), reflecting considerably different network topologies (Figures 4E,J). These results demonstrate that the coordinated expression of these genes differs between genders.

Hub genes from gender-specific modules were associated with specific cancer histological subtypes. In GC, 11 out of 12 hub genes showed over-expression in the papillary, tubular, and non-specified intestinal subtypes of males (Supplementary Figures S14A,C and Supplementary Table S6). Among these, *Hsp90ab1* and *XPO5* have been previously associated with poor prognosis and tumor-suppressor properties in GC (Melo et al., 2010; Wang et al., 2019). In TC, all 27 hub genes were predominantly over-expressed in the follicular subtype of females (Supplementary Figures S14B,D and Supplementary Table S7).

## Discussion

In this work, we set out to characterize the gender differential transcriptome in tumor and normal tissues. We selected GC and TC due to their considerable gender-biased incidence. It is well known that male and females are exposed often to very different environmental conditions (Zahm and Blair, 2003; Scarselli et al., 2018). Thus, an important limitation of this study is the lack of control for environmental effects. Despite this, we believe this analysis is still of merit since it may capture intrinsic natural variation between genders and their relation to disease susceptibility.

Our results show that SBGs in tumor and normal tissues were mostly derived from sex chromosomes, as previously found in Yuan et al. (2016). These genes are mostly common to the stomach and thyroid. Such conservation remains to be tested for other tissues. On the other hand, SBGs specific to normal or tumor tissues arose from non-sexual chromosomes, with little overlap between tissues. These results highlight the contribution of autosomes for tumor- and normal-specific sex-biased expression phenotypes, which may ultimately drive sex-biased cancer incidence.

We found metabolic processes of sterols and lipids enriched in the normal and tumor-specific SBGs of thyroid and stomach and in a female-specific module of thyroid normal tissues. Sterols are critical in signaling, regulation of lipid metabolism, development, and cellular homeostasis (Wollam and Antebi, 2011). Alterations in the metabolism of sterols and lipids are a known hallmark of cancer (Gabitova et al., 2014). Other studies have found SBGs in pathways related to the metabolism of fatty acids in cancer (Yuan et al., 2016) and in normal tissues (Naqvi et al., 2019), with a long-standing observation that genders show differences in the metabolism of lipids (Mittendorfer, 2005; Mittelstrass et al., 2011; Drolz et al., 2014). Importantly, such differences are not simply explained by the presence and action of sex hormones (Mittendorfer, 2005). Our results suggest that beyond differences in sexual hormonal regulation, the metabolic physiology of the sexes might be implicated in the gender disparity of GC and TC.

The PPAR signaling pathway was found enriched in the normal-specific SBGs of the stomach and particularly over-expressed in females. This pathway is known to control the expression of genes involved in lipid metabolism and inflammation (Varga et al., 2011), with increasing evidence that PPARα/γ inhibits tumor progression and acts as a tumor suppressor (Gou et al., 2017). Whether this finding is related to the lower GC incidence in females remains to be seen.

The normal-specific SBGs of thyroid were enriched in immune-response pathways and mostly over-expressed in females, in accordance with Naqvi et al. (2019). Thyroid hormones can trigger different responses in diverse immune cells and affect several inflammation-related processes (Jara et al., 2017). The immune system is a highly sexually dimorphic trait, with females showing an immunological advantage when facing different immune challenges (Libert et al., 2010). On the other hand, females are more prone to autoimmune diseases such as Hashimoto’s thyroiditis (HT) (Ngo et al., 2014). In the last decades, the association between HT and TC has been growing, with some studies reporting the coexistence of both diseases (Jeong et al., 2012; Zhang et al., 2012; Felicetti et al., 2017).

Altered expression of oncogenes and TSGs in normal tissue may be linked to protective or predisposing tumorigenic events (Muir and Nunney, 2015). In the stomach, females over-expressed the TSGs *FGFR3* and *ERCC2* (Lafitte et al., 2013; Zheng et al., 2015). In the thyroid, 16 of the SBGs were previously reported as cancer drivers, with 11 being over-expressed in females’ normal thyroid, including the oncogenes *CARD11*, *EZH2*, and *IL7R* (Watt et al., 2015; Kim and Roberts, 2016; Kim et al., 2018). Whether these findings are related to the differential cancer incidence between genders needs further investigation.

Tumor-specific SBGs were much less frequent, suggesting that once tumorigenesis starts, the transcriptomic differences become more diluted between genders. Of notice, in GC, we found the cancer-associated genes *LPCAT1* and *RAD51C* over-expressed in females (Meindl et al., 2010; Somyajit et al., 2010; Bi et al., 2019). In TC, among those over-expressed in males, we found *PPARG*, previously reported in thyroid carcinomas (Raman and Koenig, 2014), *ERCC5*, *MYH11*, *LEMD2*, *ZNF133*, *and IDH1*, the latter found to be mutated in thyroid carcinomas (Yang et al., 2012). *TFRC*, whose expression has been associated with poor prognosis and tumor progression (Shen et al., 2018), was found over-expressed in females. These genes are potential contributors to the gender-specific tumorigenesis of GC and TC.

Differential promoter methylation appears as a mechanism that might underlie some of the observed expression differences, given its incidence among the SBGs in both cancers and the tumor-normal differences observed for both genders in TC. Of the differentially methylated SBGs, most belong to the X-chromosome and are known to escape X-inactivation. Changes in promoter methylation in genes escaping X-inactivation have been previously found (Sharp et al., 2011). However, 46 and 80% of the SBGs in GC and TC were not differentially methylated in our study, belonging mostly to the autosomes. Moreover, half of the SBGs were not profiled by methylation probes. Other processes that contribute to sex-specific gene regulation include epigenetic regulation of enhancers (Reizel et al., 2015), estrogen-regulated miRNA expression (Klinge, 2012), and transfer RNA (tRNA) regulatory fragments (Telonis et al., 2019). Whether these processes are involved in the sex-biased patterns not explained by differential promoter methylation remains an open question.

Network analysis of co-expressed genes may help in identifying gender-specific cellular rewirings in normal and tumor tissues. Our results show that most of the network modules are preserved between genders, in agreement with previous work (Melé et al., 2015), in both types of tissues. The reasons for the different degrees and modalities of gene module preservation, in tumor and normal tissues, remain to be studied in the future. Nevertheless, we found gene modules preferentially associated for males in GC and for females in TC further associated with cancer histological subtypes.

The normal tissue surrounding the tumor can be influenced by pro-inflammatory signals released by tumors, representing an intermediate state between tumor-free healthy tissue and established neoplasms (Aran et al., 2017). Nonetheless, our analysis of the tumor-normal differential transcriptome found that a significant fraction of the DEGs were oncogenes, as previously found (Pranavathiyani et al., 2019). We also found that the most affected sex in each cancer shows an under-expression of TSGs in tumors, which is an important cancer hallmark (Hanahan and Weinberg, 2011). The same result was not observed for the opposite sex, supporting the hypothesis that TSG inactivation or deregulation may occur in the tumor tissues of the most susceptible gender. This result reinforces that genders may follow different carcinogenic programs, and therefore appropriated and differentiated therapeutic strategies may be considered (Ma et al., 2016; Yuan et al., 2016; Buoncervello et al., 2017). In TC, the female-specific tumor-normal DEGs over-expressed in normal tissues were involved in the same immune-related pathways as the male-specific DEGs over-expressed in tumors. This result may highlight some still unknown predisposing elements that make females more susceptible to TC.

In summary, we were able to identify the gender-specific expression landscape in normal and tumor tissues of the thyroid and stomach. We expect that these results provide novel insights in the understanding of the gender-differential risk underlying these cancers.

## Data Availability Statement

Publicly available datasets were analyzed in this study. This data can be found here: TCGA RNA-seq data: https://portal.gdc.cancer.gov/; GTEx RNA-seq data: https://gtexportal.org/home/; TCGA methylation data: http://firebrowse.org/.

## Author Contributions

AS, CO, and PF designed the research and wrote the manuscript. AS performed the research and analyzed the data. MF participated in the differential expression analyses design. CO and PF found funding and infrastructures for the work. All authors revised the manuscript.

## Conflict of Interest

The authors declare that the research was conducted in the absence of any commercial or financial relationships that could be construed as a potential conflict of interest.

## Abbreviations

DEGs: differentially expressed genes
GC: gastric cancer
GTEx: Genotype-Tissue Expression
SBGs: sex-biased genes
TC: thyroid cancer
TCGA: The Cancer Genome Atlas
TSGs: tumor-suppressor genes.
