# Supplementary material for: Gender Differential Transcriptome in Gastric and Thyroid Cancers
Source: Front Genet. 2020 Jul 30;11:808. doi: 10.3389/fgene.2020.00808 (PMC7406663; doi:10.3389/fgene.2020.00808)
Supplement: Supplementary file 1 [file Data_Sheet_1.pdf]

Supplementary methods

Data collection

We obtained TCGA mRNA-seq and clinical data for gastric cancer (GC) and thyroid cancer (TC) tumour matched-normal samples. The data was downloaded from the GDC data portal (https://portal.gdc.cancer.gov/) in RPKM and read counts formats, at the gene-level. Additional clinical information was obtained from (Cancer Genome Atlas Research Network, 2014a); (Cancer Genome Atlas Research Network, 2014b); (Liu et al., 2018). The TCGA methylation data was acquired from the FireBrowse portal (http://firebrowse.org/), as beta values per methylation probe (450k arrays). We also compiled GTEx v6 mRNA-seq and phenotypic data, for stomach and thyroid normal samples, from the GTEx portal (https://gtexportal.org/home/) in RPKM and read counts formats. The gene annotation was downloaded from the GDC portal for the TCGA mRNA-seq data (GENCODE v22) and from the GENCODE website (https://www.gencodegenes.org/) for the GTEx mRNA-seq data (GENCODE v19).

A list of human tumour-suppressor genes (TSGs) (Zhao et al., 2013) and oncogenes (Liu et al., 2017) were downloaded from https://bioinfo.uth.edu/TSGene/ and http://ongene.bioinfo-minzhao.org/index.html, respectively. The Cancer Gene Census catalogue (Sondka et al., 2018) was downloaded from https://cancer.sanger.ac.uk/census. A list of cancer driver genes was downloaded from (Bailey et al., 2018). The X-chromosomal genes known to escape inactivation were obtained from (Tukiainen et al., 2017).

Data pre-processing

We assembled the TCGA mRNA-seq (read counts and RPKMs) and clinical data in tabular formats using in-house scripts. The datasets comprised 60483 genes across 407 samples (375 primary tumours and 32 matched-normal) for GC and 560 samples (502 primary tumours and 58 matched-normal) for TC. For downstream analysis we selected only the protein-coding and lincRNA genes, comprising 27470 genes, as described in GENCODE v22 annotation. In order to remove lowly-expressed genes, we filtered out those without 5 counts-per-million (CPM) in at least 20% of the tumour or normal samples. After gene filtering, the mRNA-seq datasets comprised 12690 genes for TC and 13674 genes for GC.

The GTEx mRNA-seq datasets (read counts and RPKMs) comprised 56318 genes, across 193 samples for stomach and 323 samples for thyroid tissues. After selecting the protein-coding and lincRNA genes (27459 genes), as described in GENCODE v19 annotation, we removed those genes without 5 CPM in at least 20% of samples. The final mRNA-seq datasets comprised 12501 genes for thyroid and 12371 genes for stomach tissues. The CPM values were calculated using the *cpm* function from the edgeR package (Robinson et al., 2010).

After merging the TCGA and GTEx samples in each tissue, the final mRNA-seq datasets comprised 11734 genes for thyroid and 11842 genes for stomach. A PCA analysis was then performed using *prcomp* function in R.

We regressed-out potential confounding covariates from the GTEx gene expression data (log2 RPKM) using the following multiple linear model:

[1] $g_i = \boldsymbol{\beta}_0 + \boldsymbol{\beta}_1 smrin + \boldsymbol{\beta}_2 age + \boldsymbol{\beta}_3 ethncty + \boldsymbol{\beta}_4 mhcancernm + \boldsymbol{\beta}_5 smcenter + \boldsymbol{\beta}_6 smtstptref + \boldsymbol{\beta}_7 smnabtcht + \boldsymbol{\beta}_8 smtsisch + \varepsilon$

where g_i represents the gene expression for gene i, $\boldsymbol{\beta}_0$ the intercept, $\boldsymbol{\beta}_i$ $i \in (1, ..., 8)$, the regression coefficients for the covariates, and ε the noise term. See **table S8** for additional information about the covariates. The gene expression corrected for these covariates corresponded to the residuals of this model, calculated as:

[2] $g_i' = g_i - \hat{g}_i$

where g_i' represents the gene expression corrected for these covariates, g_i the observed gene expression and $\hat{g}_i$ the predicted gene expression from the model. The linear models were calculated using the *lm* R function.

Differential gene expression

We performed the differential expression analyses using the edgeR package. edgeR models the variance of the read counts per gene using a negative binomial distribution, and applies a generalized linear model (GLM) to account for additional covariates when testing for differential expression.

We performed differential gene expression between genders in TCGA tumour and GTEx normal samples from stomach and thyroid. We also performed differential gene expression between TCGA tumours and matched-normal samples in each gender. In each comparison we created a design matrix taking into account several covariates. In R notation:

Male vs female in TCGA tumour samples:
[3] *design = model.matrix(~ race + ethnicity + age + tumor stage + histology + tss + portion + plate + gender, data = covars.matrix)*

Male vs female in GTEx normal samples:
[4] *design = model.matrix(~ smrin + age + ethncty + mhcancernm + smcenter + smtstptref + smnabtcht + smtsisch + gender, data = covars.matrix)*

Tumour vs matched-normal TCGA samples in each sex:
[5] *design = model.matrix(~ race + ethnicity + age + tss + portion + plate + tissue type, data = covars.matrix)*

where *design* corresponds to the design matrix, *model.matrix* the R function used to define the design matrices, each term (e.g. *age*) the respective covariate, and *covars.matrix* the data frame containing the covariates. See **Table S8** for additional information about the covariates. In each comparison we normalized the read counts using the trimmed-mean of M-values method (Robinson and Oshlack, 2010), with the *calcNormFactors* function. After estimating the common and tagwise dispersions with *estimateDisp*, we fitted a GLM model for each gene using *glmFit*. A likelihood ratio test (LRT) was then applied on the coefficients of tissue type (tumour or normal) or gender (male or female) to test for differences between these samples, using the *glmLRT* function. The P-values were adjusted for false discovery rate using the Benjamini-Hochberg procedure.

We selected the differentially expressed genes between genders (sex-biased genes [SBGs]) using an FDR lower than 5%, additionally requiring for the differentially expressed genes (DEGs) between tumour and matched-normal samples an absolute log2 fold-change higher than 1. Tumour and normal-specific SBGs were calculated by intersecting both gene sets. The same process was performed to calculate male- and female-specific DEGs between tumour and matched-normal samples.

We also performed a differential expression analysis between genders using the TCGA normal samples, adjusted for race, ethnicity, age and portion (**Table S8**). In this analysis we found 11 and 26 SBGs in stomach and thyroid, respectively, and only two normal-specific SBGs in both tissues. The relative low number of SBGs, alongside the higher number of samples in the GTEx cohort, led us to consider the GTEx dataset for the further analyses in this paper. We rationale that the high number of samples in GTEx would help to robustly quantify the gender differential transcriptome.

We also used limma (with voom) differential expression method (Law et al., 2014), which assumes a normal distribution for the gene expression data, instead of a negative binomial distribution as edgeR. We found an overlap greater than 85% with edgeR (in all comparisons), indicating a robust set of called differentially expressed genes.

Differential gene promoter methylation

We selected TCGA methylation probes annotated to gene promoter regions using the R package IlluminaHumanMethylation450kanno.ilmn12.hg19. Then, for each gene in each sample, we calculated the average beta value across the promoter probes.

The differential gene promoter methylation analysis was performed using a Wilcoxon rank-sum test (*wilcox.test* R function). We assessed differences on gene promoter methylation between genders in TC and GC, and between tumour and matched-normal samples in TC, independently for each gender. The GC matched-normal samples were not profiled by the selected methylation array, hampering the tumour-normal differential methylation analysis in GC. The P-values were adjusted for false discovery rate using the Benjamini-Hochberg procedure. Genes with FDR < 5% were defined as differentially methylated.

In GC and TC, 56% of the genes with expression data were covered by methylation probes. For the SBGs, 51% in GC and 48% in TC contained information about the differential

methylation status (**Figure S7A, S7B**). For the tumour-normal DEGs in TC, 30% in females and 34% in males were profiled by differential methylation (**Figure S7C, S7D**).

Construction of gene co-expression networks

We built gene co-expression networks for each gender in TCGA tumour and GTEx normal samples from stomach and thyroid, using the methods in the WGCNA package (Langfelder and Horvath, 2008). First, we log2 transformed and transposed the RPKM matrices. For the GTEx samples we used the gene expression data after regressing-out the confounding covariates. Then, we removed potential outlier samples using a hierarchical clustering dendrogram (*hclust* R function, using *average* as agglomeration method). The distances between samples were calculated using the *euclidean* distance measure, using the *dist* function. In GC we removed 8 samples in females *(cut height = 117)* and 4 samples in males *(123)*. In TC we removed 3 samples in females *(120)* and 4 samples in males *(100)*, while in thyroid normal tissue we removed 3 samples in females *(70)* and 1 sample in males *(80)*.

Network construction

In order to build gene co-expression networks we calculated an adjacency matrix using the following expression:

[6] $a_{ij} = |corr(g_i, g_j)|^{\beta}$

where a_{ij} corresponds to the connection strength between gene i (g_i) and gene j (g_j), |corr| the absolute Pearson's correlation coefficient, and β the soft-thresholding power that approximates the network of a scale-free topology. Raising the absolute correlation values to a power accentuates high correlations at the expense of low correlations.

Module detection

After network construction the next step was to find gene modules or clusters of densely interconnected genes. For that, we converted the adjacency matrix into a topological overlap matrix (TOM). The TOM contains the pairwise relative interconnectedness of all nodes in the network. After converting the TOM into a dissimilarity measure (1 - TOM), we identified gene modules by cutting off the branches of a hierarchical clustering dendrogram (hclust R function using *average* as agglomeration method). The branches were cut using the Dynamic Hybrid algorithm (Langfelder et al., 2008). This method eliminates the need of using constant height cutoff values and is more effective in complex dendrograms. The gene expression profiles of a module can be summarized by its module eigengene (first principal component). We merged highly similar modules if the eigengene Pearson's correlation was higher than 0.75. Genes without module assignment were not considered for further analyses.

We performed the network construction and module detection steps automatically and sequentially, using the *blockwiseModules* function. We used the parameters as shown by the

authors in the WGCNA tutorials (tutorial I, section 2.a.) (https://horvath.genetics.ucla.edu/html/CoexpressionNetwork/Rpackages/WGCNA/Tutorials/). The only exceptions were the parameters *maxBlockSize*, *power* and *minModuleSize*. In order to perform the network construction in a single gene block we increased the maximum block size (*maxBlockSize*) from 5000 (the default) to 20000. This prevents the network module assignments from being split into multiple blocks. We estimated the optimal powers that approximate the networks of a scale-free topology, using the *pickSoftThreshold* function. Following WGCNA recommendations, we selected the highest power that exceeds the scale-free topology fit R^2 cutoff, set to 0.85 by default. In thyroid the powers were set to 9 for all networks, except for the males network in tumours, whose power was set to 10. For stomach the powers were set to 4 for all networks. Instead of 30 genes as minimum module size (*minModuleSize*), we opted to keep the default value of 20 genes. Using this approach we built 8 networks in total.

Gender differential co-expression network analysis

We compared male to female networks using a strategy based on (Melé et al., 2015). We started by computing the percentage of gene content overlap between each pair of modules, where each module belongs to a different network. As an example, given the modules M and F in the males and females network, respectively, we calculated the overlap between M and F as follows:

[7] $overlap_{MF} = \frac{|M \cap F|}{min(M,F)} \times 100$

where $overlap_{MF}$ corresponds to the percentage of overlap between M and F, $|M \cap F|$ is the number of genes in common between M and F, and min(M, F) is the length of the smaller module. We also calculated a Fisher's exact test P-value for each overlap, using the *overlapTable* function from the WGCNA package. Based on the P-value and on $overlap_{MF}$, we considered the overlap of a given pair of modules as:

- *absent*, if P-value > 5% or (P-value < 5% and $overlap_{MF}$ < 20%);
- *low*, if P-value < 5% and (20% ≤ $overlap_{MF}$ < 50%);
- *moderate*, if P-value < 5% and (50% ≤ $overlap_{MF}$ < 70%);
- *high*, if P-value < 5% and ($overlap_{MF}$ ≥ 70%).

For both genders the modules were classified as *lowly*, *moderately* or *highly*-preserved, if the overlap with the opposite gender has been defined as *low*, *moderate* or *high*, respectively. We honoured the highest overlap when multiple overlaps occurred. As an example, in the stomach normal networks from GTEx, the largest module from males (5976 genes) has a *high* and a *moderate* overlap with two modules from females (with 3903 and 936 genes, respectively) (**Figure S12B**). Therefore, we considered the male module as highly-preserved

in females. Modules without overlap with the modules of the opposite gender were classified as gender-specific.

Association of gender-specific modules with cancer clinical traits

The biological significance of a module can be defined as the absolute correlation between the module eigengene *E* and a sample phenotype *P* (Langfelder and Horvath, 2008). Modules with high biological significance (correlation) can represent pathways associated with the phenotype *P*. We evaluated the biological significance of the gender-specific modules in tumours by fitting a linear regression model as follows:

[8] $E = \boldsymbol{\beta}_0 + \boldsymbol{\beta}_1 P + \varepsilon$

where *E* represents the module eigengene, $\boldsymbol{\beta}_0$ the intercept, ε the noise term and *P* the cancer clinical traits overall survival (in days), tumour stage (AJCC staging system) and cancer histological subtype. In TC, we considered the cancer histological subtypes classical (number of samples: 257), follicular (75) and tall cell (26). In GC, the cancer subtypes signet ring (9), diffuse (36), intestinal mucinous (14), intestinal NOS (not otherwise specified) (45), intestinal papillary (3) and intestinal tubular (39). The association between the modules and the clinical traits was then evaluated using the regression-derived R^2 and the one-way ANOVA P-values. The linear models were calculated using the *lm* R function. We also evaluated the association between survival and the modules eigengene using univariate cox proportional hazard regression models. These models were computed using the *coxph* function for the survival R package.

In a given module related with phenotypic traits, the hub genes (highly connected) are the most relevant genes to look for, since their expression profiles can represent that of the entire module (Langfelder and Horvath, 2008). In the gender-specific modules associated with the cancer histological subtypes, we investigated the cancer subtypes where these genes are predominantly expressed. For that we selected the hub genes of each module (with absolute intramodular connectivity [$|K_{ME}|$] > 0.8) and tested them for differential expression between cancer histological subtypes, using a Kruskal-Wallis rank sum test (*kruskal.test* R function).

Functional enrichment analysis

We performed functional enrichment using hypergeometric tests and gene set enrichment analysis (GSEA), implemented in the functions *enrichr* and *GSEA* from the clusterProfiler R package (Yu et al., 2012). We used gene sets downloaded from the MSigDB database (http://software.broadinstitute.org/gsea/msigdb), including C1 positional sets, C2 KEGG pathways and C5 GO biological processes (BP). We applied GSEA on gene modules derived from gene co-expression networks, sorted by K_{ME}. The comparison of enrichment profiles between gender-specific DEGs over-expressed in tumour or normal tissues was

performed using the hypergeometric test implemented in the function *compareCluster*. The P-values were adjusted for false discovery rate using the Benjamini-Hochberg procedure.

The enrichment for TSGs, oncogenes, X-chromosomal genes escaping inactivation and differentially methylated genes was performed using a Fisher's exact test *(fisher.test* R function; *alternative = "greater")*.

The backgrounds corresponded to all genes that were analysed in our study, either by differential expression or by gene co-expression networks.

All gene lists reported in this study were annotated with functional gene summaries, using the function *queryMany* from the mygene R package.

Code availability

The computational analyses were performed in R 3.6.3 and all the code is available under a GNU General Public License V3 in a GitHub project, at the following url: https://github.com/abelfsousa/gender_differences. The differential expression analyses were performed with edgeR 3.26.8 and the gene co-expression network analyses with WGCNA 1.68. The functional enrichment analysis (hypergeometric tests and GSEA) were performed using clusterProfiler 3.12.0. Plotting was done using ggplot2 3.2.1, ComplexHeatmap 2.0.0, arcdiagram 0.1.12 and eulerr 6.1.0. Data analysis and structuring using dplyr 0.8.3, tidyr 1.0.0 and the remaining packages included in tidyverse 1.2.1.

References

Bailey, M. H., Tokheim, C., Porta-Pardo, E., Sengupta, S., Bertrand, D., Weerasinghe, A., Colaprico, A., Wendl, M. C., Kim, J., Reardon, B., et al. (2018). Comprehensive characterization of cancer driver genes and mutations. *Cell* 173, 371-385.e18. doi:10.1016/j.cell.2018.02.060.

Cancer Genome Atlas Research Network (2014a). Comprehensive molecular characterization of gastric adenocarcinoma. *Nature* 513, 202–209. doi:10.1038/nature13480.

Cancer Genome Atlas Research Network (2014b). Integrated genomic characterization of papillary thyroid carcinoma. *Cell* 159, 676–690. doi:10.1016/j.cell.2014.09.050.

Langfelder, P., and Horvath, S. (2008). WGCNA: an R package for weighted correlation network analysis. *BMC Bioinformatics* 9, 559. doi:10.1186/1471-2105-9-559.

Langfelder, P., Zhang, B., and Horvath, S. (2008). Defining clusters from a hierarchical cluster tree: the Dynamic Tree Cut package for R. *Bioinformatics* 24, 719–720. doi:10.1093/bioinformatics/btm563.

Law, C. W., Chen, Y., Shi, W., and Smyth, G. K. (2014). voom: Precision weights unlock linear model analysis tools for RNA-seq read counts. *Genome Biol.* 15, R29. doi:10.1186/gb-2014-15-2-r29.

Liu, J., Lichtenberg, T., Hoadley, K. A., Poisson, L. M., Lazar, A. J., Cherniack, A. D., Kovatich, A. J., Benz, C. C., Levine, D. A., Lee, A. V., et al. (2018). An Integrated TCGA Pan-Cancer Clinical Data Resource to Drive High-Quality Survival Outcome Analytics. *Cell* 173, 400-416.e11. doi:10.1016/j.cell.2018.02.052.

Liu, Y., Sun, J., and Zhao, M. (2017). ONGene: A literature-based database for human oncogenes. *J. Genet. Genomics* 44, 119–121. doi:10.1016/j.jgg.2016.12.004.

Melé, M., Ferreira, P. G., Reverter, F., DeLuca, D. S., Monlong, J., Sammeth, M., Young, T. R., Goldmann, J. M., Pervouchine, D. D., Sullivan, T. J., et al. (2015). The human transcriptome across tissues and individuals. *Science* 348, 660–665. doi:10.1126/science.aaa0355.

Robinson, M. D., McCarthy, D. J., and Smyth, G. K. (2010). edgeR: a Bioconductor package for differential expression analysis of digital gene expression data. *Bioinformatics* 26, 139–140. doi:10.1093/bioinformatics/btp616.

Robinson, M. D., and Oshlack, A. (2010). A scaling normalization method for differential expression analysis of RNA-seq data. *Genome Biol.* 11, R25. doi:10.1186/gb-2010-11-3-r25.

Sondka, Z., Bamford, S., Cole, C. G., Ward, S. A., Dunham, I., and Forbes, S. A. (2018). The COSMIC Cancer Gene Census: describing genetic dysfunction across all human cancers. *Nat. Rev. Cancer* 18, 696–705. doi:10.1038/s41568-018-0060-1.

Tukiainen, T., Villani, A.-C., Yen, A., Rivas, M. A., Marshall, J. L., Satija, R., Aguirre, M.,

Gauthier, L., Fleharty, M., Kirby, A., et al. (2017). Landscape of X chromosome inactivation across human tissues. *Nature* 550, 244–248. doi:10.1038/nature24265.

Yu, G., Wang, L.-G., Han, Y., and He, Q.-Y. (2012). clusterProfiler: an R package for comparing biological themes among gene clusters. *OMICS* 16, 284–287. doi:10.1089/omi.2011.0118.

Zhao, M., Sun, J., and Zhao, Z. (2013). TSGene: a web resource for tumor suppressor genes. *Nucleic Acids Res.* 41, D970-6. doi:10.1093/nar/gks937.
