# Supplementary material for: Gender Differential Transcriptome in Gastric and Thyroid Cancers
Source: Front Genet. 2020 Jul 30;11:808. doi: 10.3389/fgene.2020.00808 (PMC7406663; doi:10.3389/fgene.2020.00808)
Supplement: Supplementary file 2 [file Data_Sheet_2.PDF]

Supplementary figures

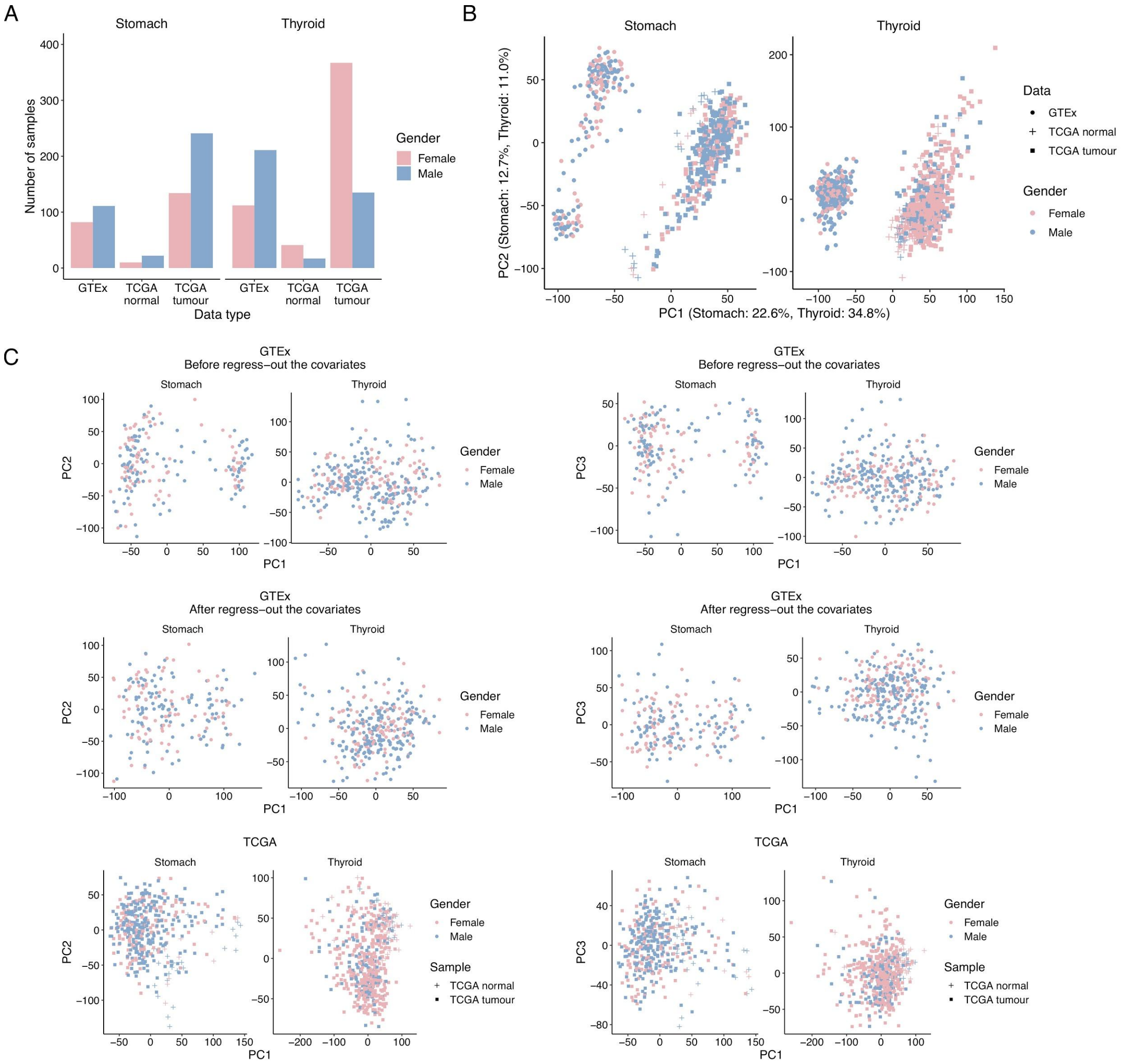

Supplementary figure 1. Number of samples and PCA analysis. (A) Number of samples by gender and data type (GTEx, TCGA tumour and TCGA normal) for stomach and thyroid. **(B)** Scatter plot representing the PCA analysis (each dot is a sample). The dot shape represents the data type and the color represents the gender. PC1 explains most of the variance and separates GTEx from TCGA samples. **(C)** Confounding effects regressed-out from the GTEx RNA-seq data (**Supplementary Methods**). The top plots show the PC1 *vs* PC2 (left) and PC1 *vs* PC3 (right) before regressing-out the covariates. The middle plots show the same data after regressing-out the covariates. The TCGA RNA-seq data (bottom plots) was not regressed-out, as the PCA did not reveal a clear sample separation.

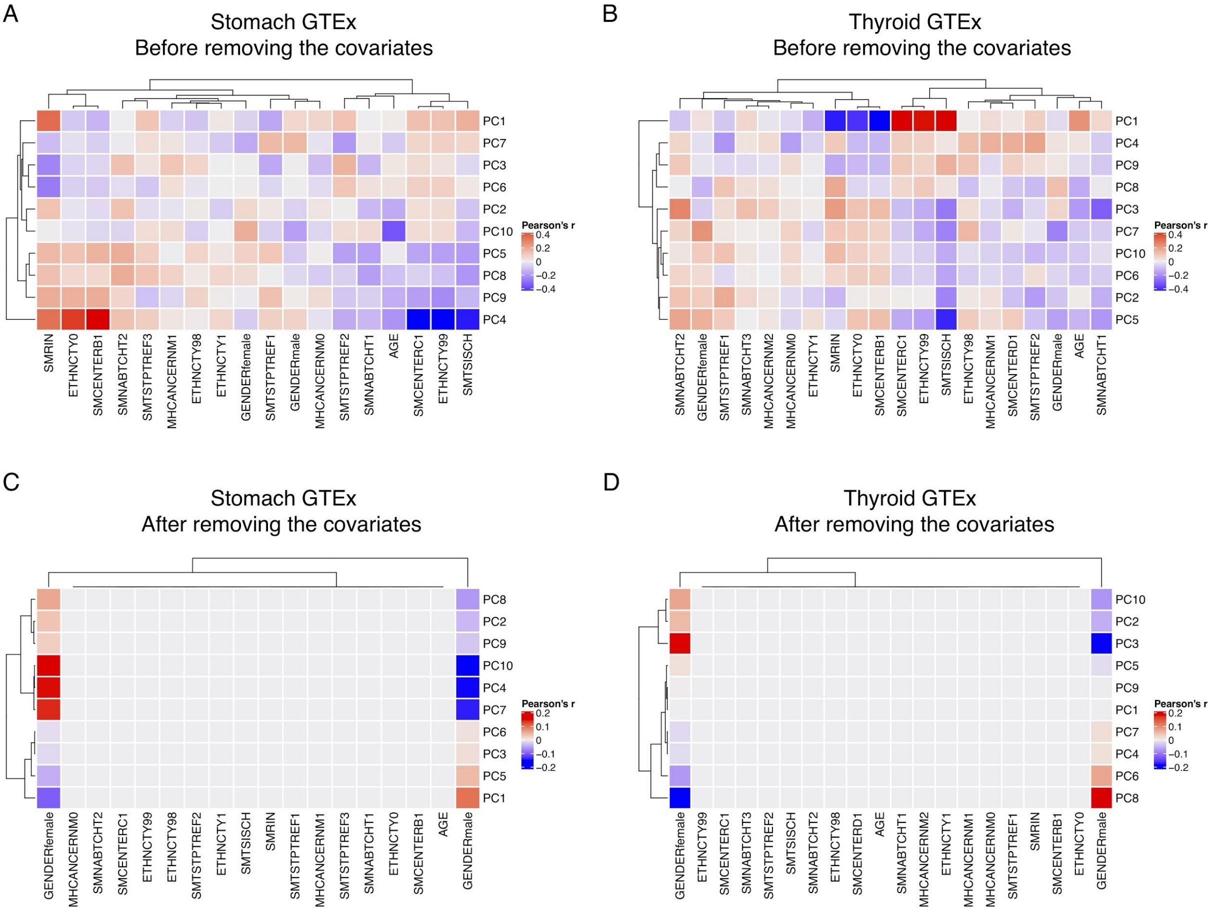

Supplementary figure 2. Pearson correlation of the first 10 principal components (PCs) from PCA with covariates before and after normalization. Stomach and thyroid before **(A)** **(B)** and after removing the covariate effect **(C)** **(D)**. The PCA was performed using the GTEx gene expression data (log2 FPKM). Categorical covariates were converted into binary variables. Each level is represented in the heatmap (columns). The gender covariate was not regressed-out. See **Table S8** for information about the covariates and **Supplementary methods** for details about the model used.

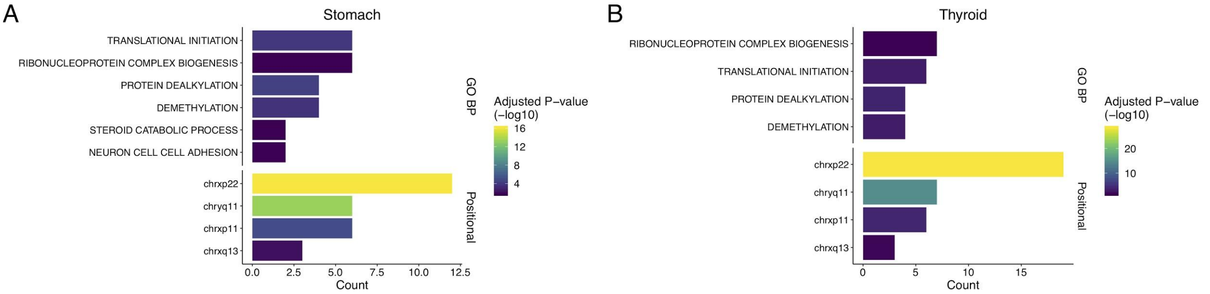

Supplementary figure 3. Functional enrichment analysis on the SBGs shared by the normal and tumour tissues. GO biological processes (GO BP) and genomic positions (Positional) enriched in stomach **(A)** and in thyroid **(B)** (top 5; FDR < 5%).

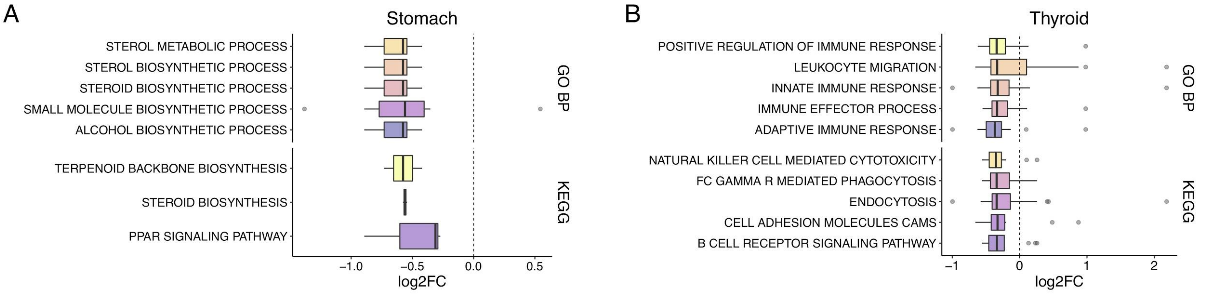

Supplementary figure 4. Distribution of gene fold-changes (log2) for the enriched terms in the normal-specific SBGs (related to the main Figures 2G and 2H). (A) is for stomach and **(B)** is for thyroid. To the left of the vertical lines are the genes over-expressed in females and to the right the genes under-expressed in females (over-expressed in males).

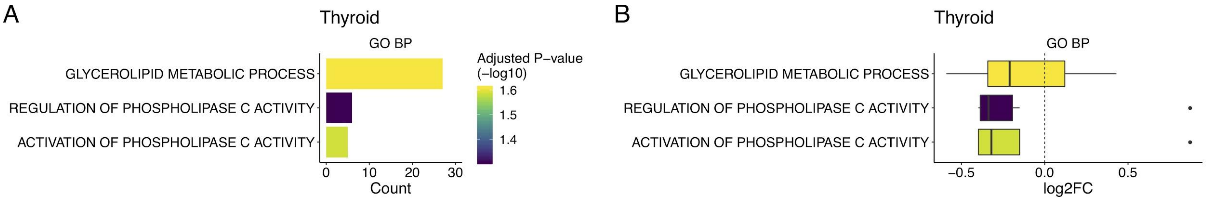

Supplementary figure 5. GO biological processes (GO BP) related to lipids metabolism enriched in the thyroid normal-specific SBGs. (A) GO BP terms enriched (FDR < 5%). **(B)** Distribution of gene fold-changes (log2) for the enriched terms. To the left of the vertical lines are the genes over-expressed in females and to the right the genes under-expressed in females (over-expressed in males).

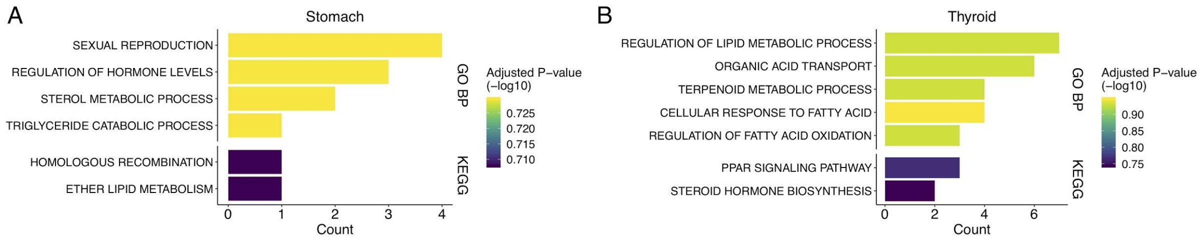

Supplementary figure 6. Functional enrichment analysis on the tumour-specific SBGs. GO biological processes (GO BP) and KEGG pathways enriched in stomach **(A)** and in thyroid **(B)** (top 5; FDR < 20%).

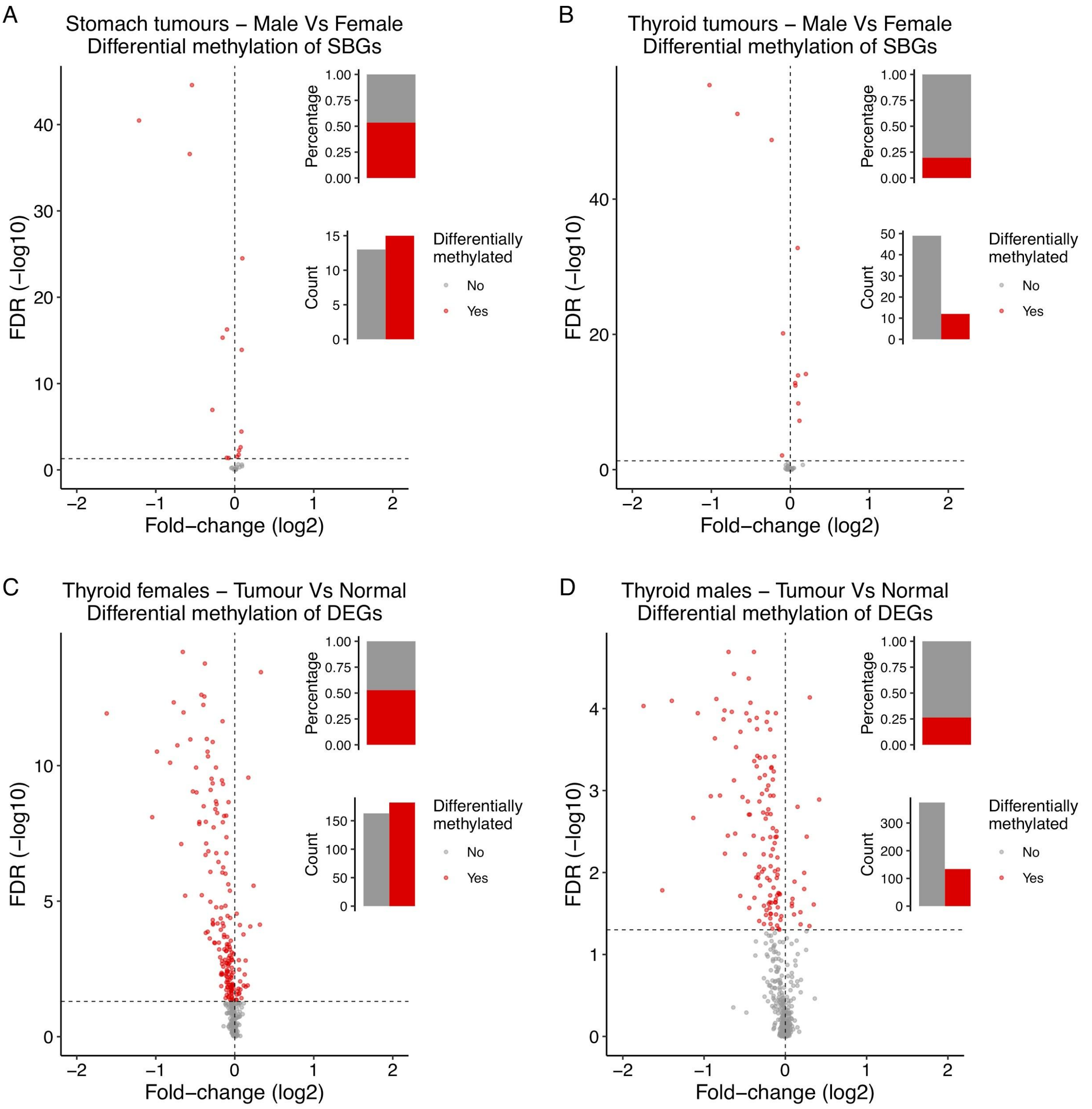

Supplementary figure 7. Differential promoter methylation analysis. Only the differentially expressed genes (gender-biased [SBGs] and tumour-normal [DEGs]) with information on differential methylation status are represented. (A) (B) Differential

methylation status of the SBGs in GC and TC, respectively. Genes with FDR < 5% were defined as differentially methylated. **(C) (D)** Differential methylation status of the tumour-normal DEGs in TC for females and males, respectively. Genes with FDR < 5% were defined as differentially methylated. No methylation data (beta values per probe) was available for the tumour-matched normal samples of GC to perform the same analysis. The barplots in the middle and upper right corners show the number and percentage of differentially methylated genes, respectively. All differentially expressed gene sets showed an enrichment for differentially methylated genes in comparison to the background (genes not differentially expressed and with information on differential methylation status). P-value = 2.0e-15, 2.7e-6, 1.0e-3 and 1.7e-24, for (A), (B), (C) and (D), respectively.

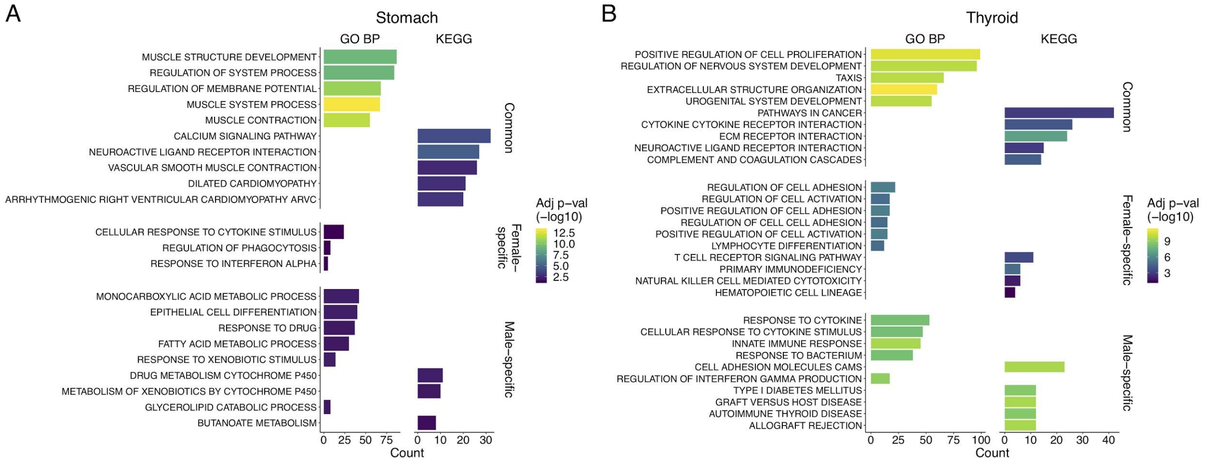

Supplementary figure 8. Functional enrichment analysis on the tumour-normal DEGs. GO biological processes (GO BP) and KEGG pathways enriched in stomach **(A)** and in thyroid **(B).** The terms are represented by DEG group: common, female-specific and male-specific (top 5; FDR < 5%).

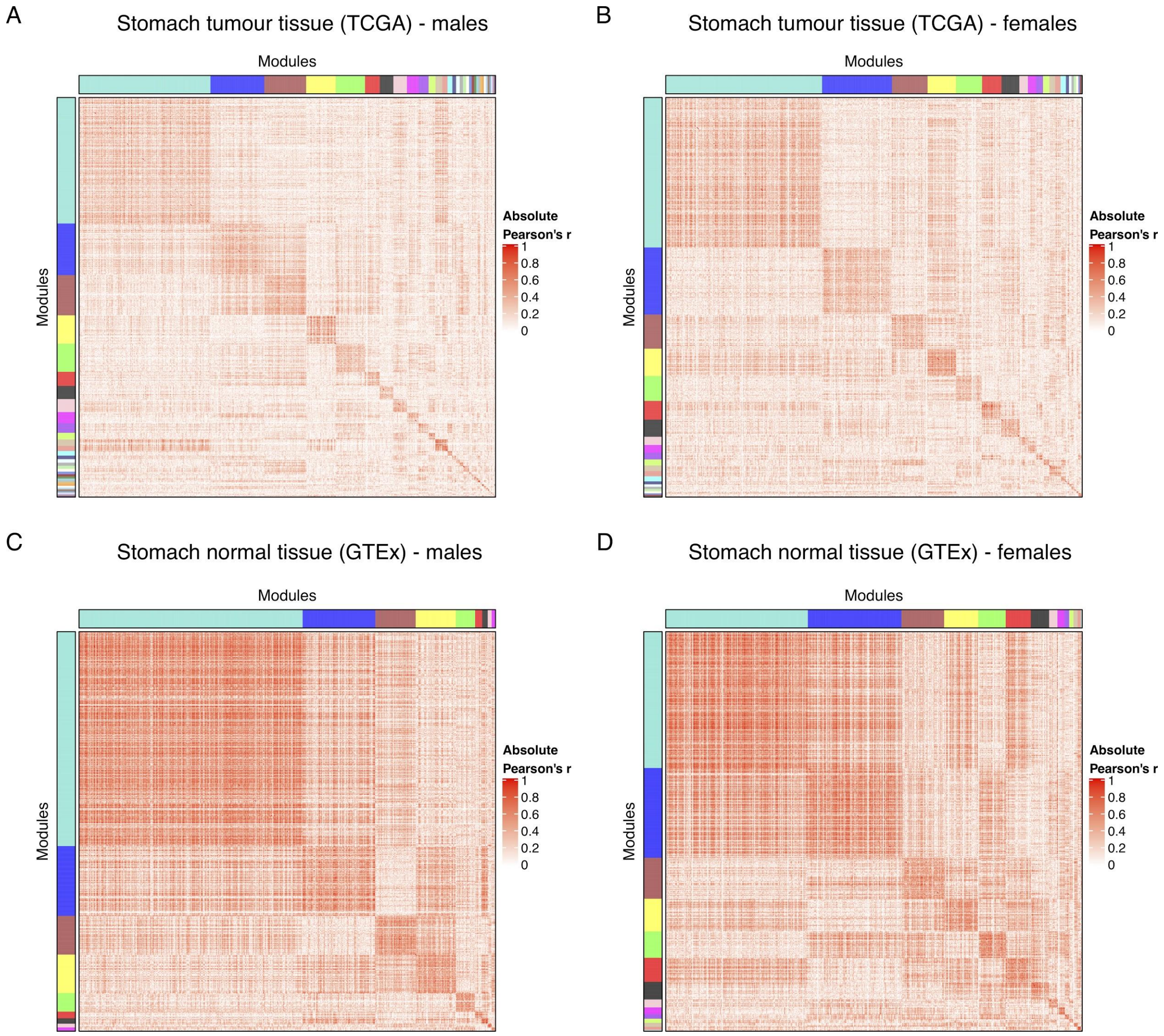

Supplementary figure 9. Correlation matrix of the WGCNA networks for the stomach tissue. Absolute pearson correlation between gene expression profiles (log2 FPKM). The gene module assignments are shown by the color bars at the top and left of each plot. Network representation for the tumour tissue of males **(A)** and females **(B)** and for the normal tissue of males **(C)** and females **(D)**.

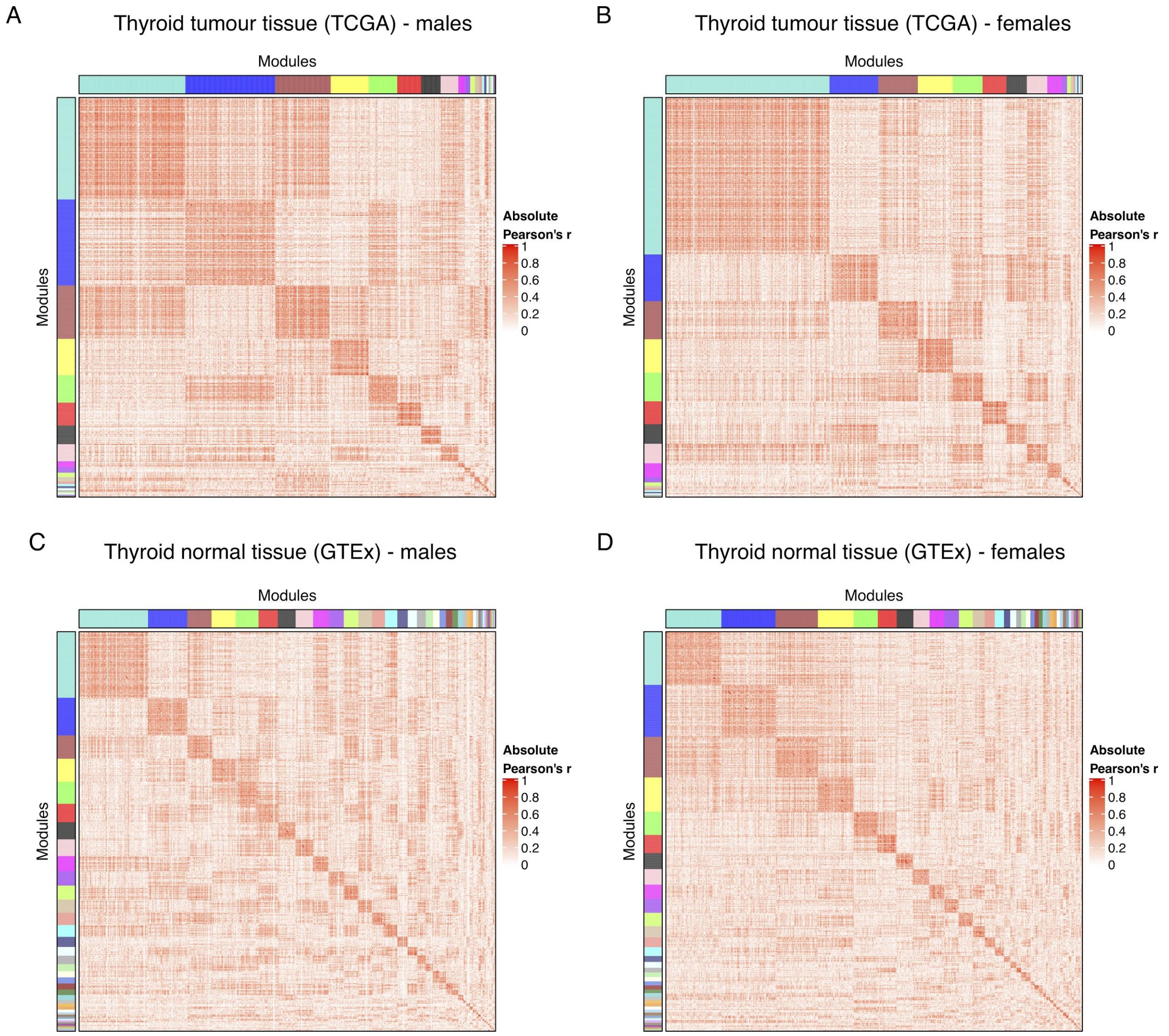

Supplementary figure 10. Correlation matrix of the WGCNA networks for the thyroid tissue. Absolute pearson correlation between gene expression profiles (log2 FPKM). The gene module assignments are shown by the color bars at the top and left of each plot. Network representation for the tumour tissue of males **(A)** and females **(B)** and for the normal tissue of males **(C)** and females **(D)**.

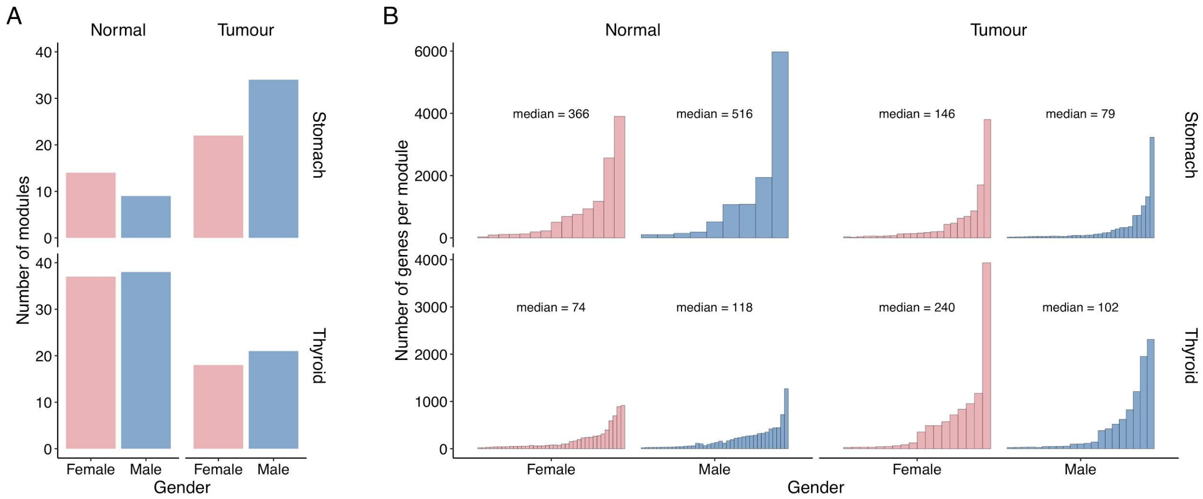

Supplementary figure 11. Features of gender co-expression networks in normal and tumour tissues of stomach and thyroid. (A) Number of gene modules in each network. **(B)** Number of genes across gene modules.

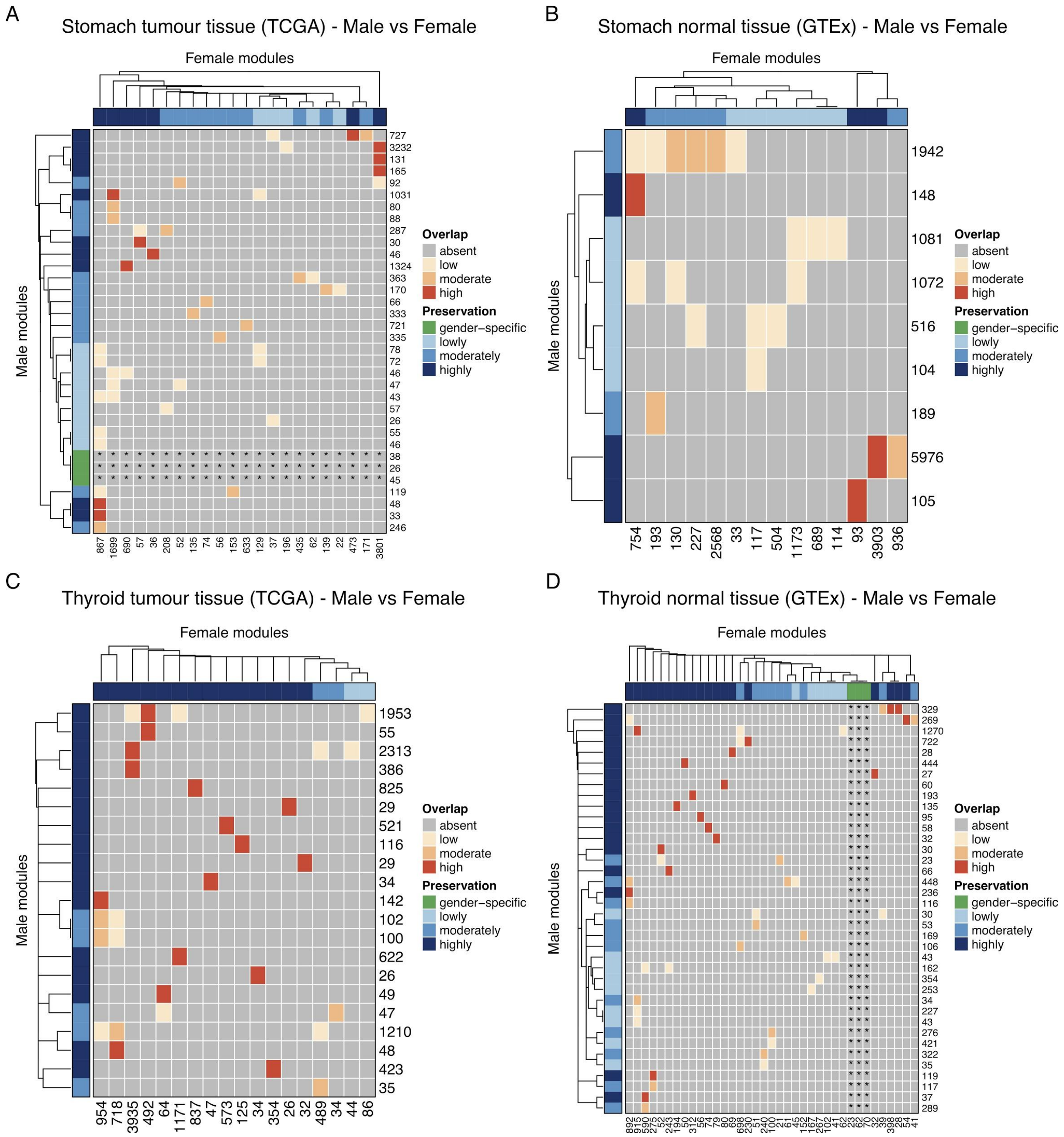

Supplementary figure 12. Comparison of modules between genders in the stomach and thyroid tissues. **(A)** and **(B)** are for the tumour and normal tissues of stomach; **(C)** and **(D)** are for the tumour and normal tissues of thyroid. Male modules are in the rows and females modules are in the columns. The numbers in the rows and columns are the number of genes inside each module. The *Overlap* legend represents the degree of gene content overlap between genders (**Supplementary Methods**). The *Preservation* legend indicates the preservation of the modules in the opposite gender, based on the degree of overlap. Gender-specific modules are marked with asterisks.

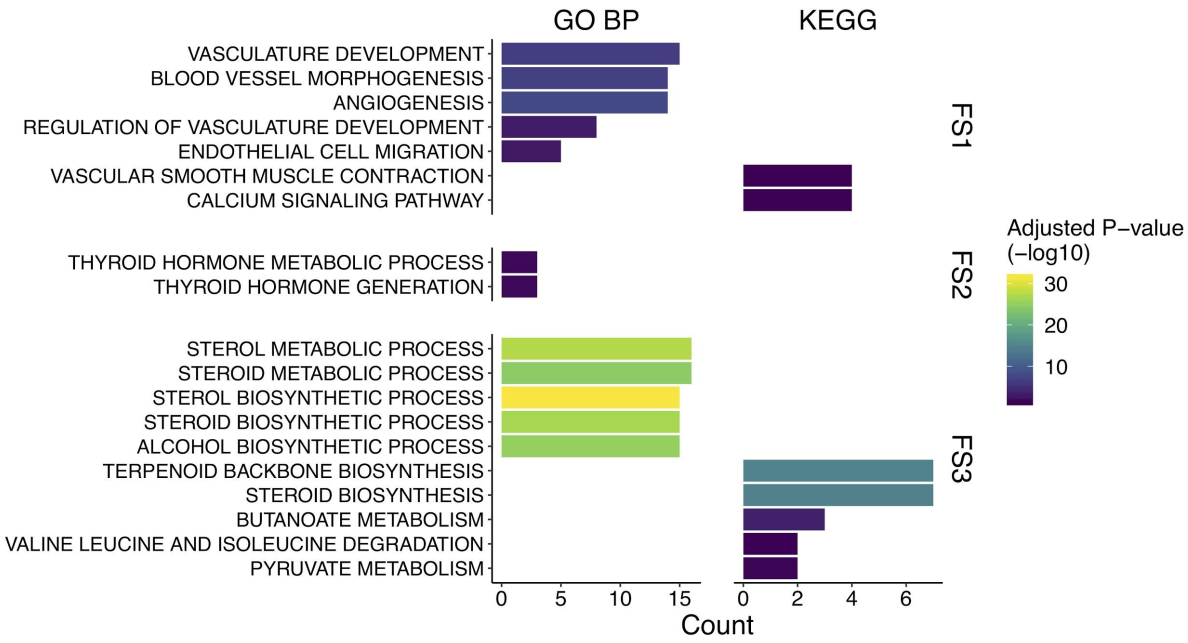

Supplementary figure 13. Functional enrichment analysis of the female-specific modules in thyroid normal tissues. GO biological processes (GO BP) and KEGG pathways. The terms are represented for the female-specific modules FS1, FS2 and FS3 (top 5; FDR < 5%).

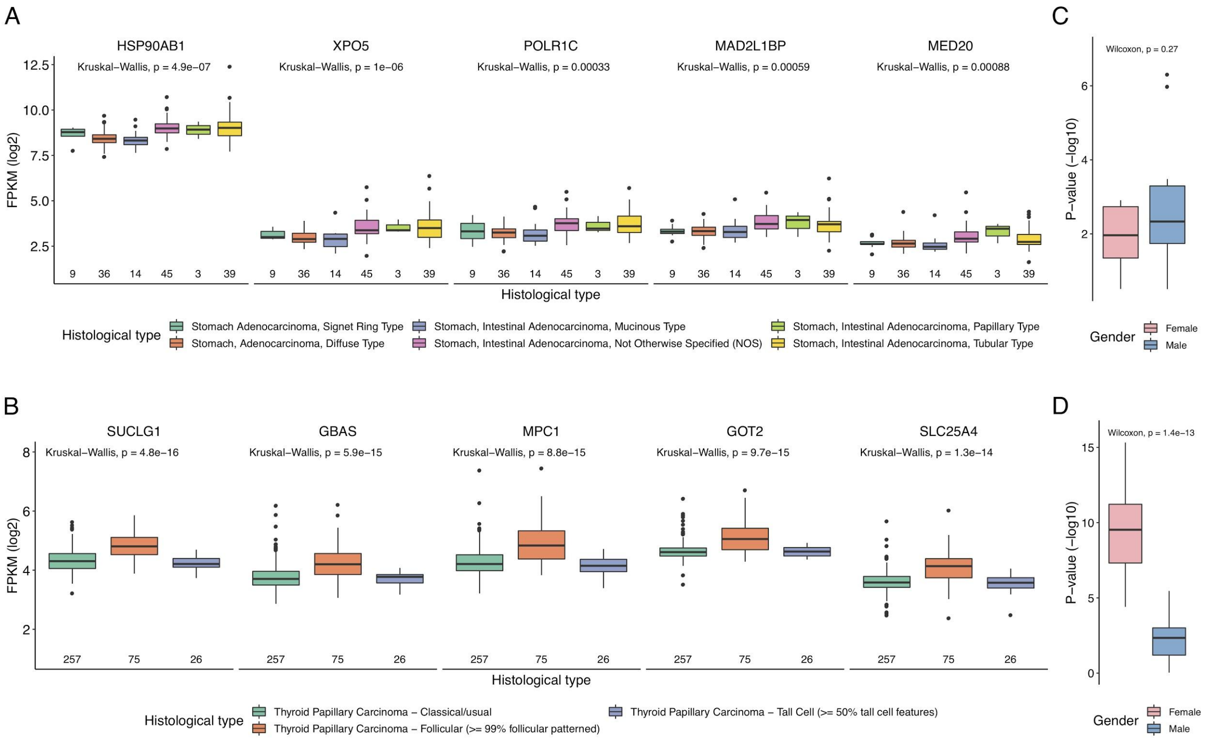

Supplementary figure 14. Differential expression between cancer histological subtypes of hub genes from gender-specific modules. (A) (B) Hub genes differentially expressed between cancer histological subtypes, in males for GC and in females for TC, respectively (top 5; *P-value < 0.05*). The number of samples is underneath each box. The Kruskal-Wallis rank sum test P-value is shown. **(C) (D)** Comparison of the Kruskal-Wallis P-value distribution between genders. The Wilcoxon rank-sum test P-value is shown. The median of the distribution is higher for males in GC and for females in TC, the genders where the modules tend to be specific.
